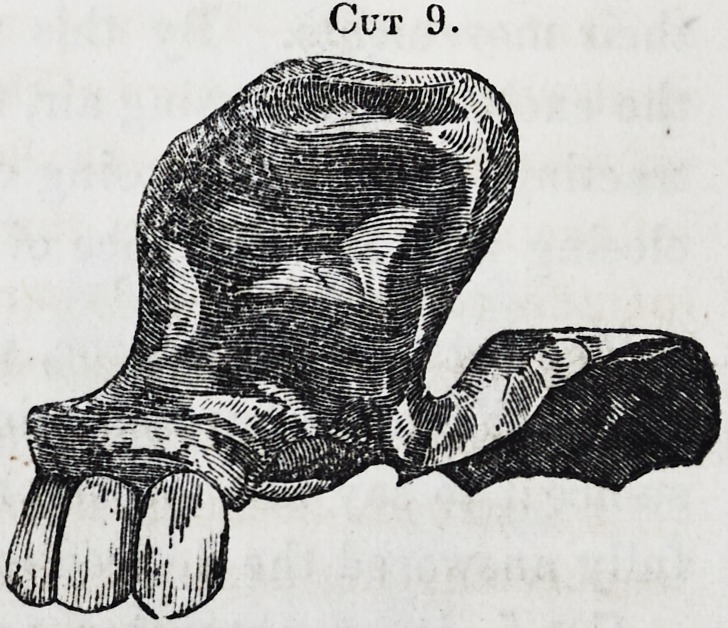# Dental Obturators

**Published:** 1855-01

**Authors:** 


### ARTICLE V.

### *Dental Obturators.* By A. A. B.

IN view of the subject designated for this paper being confined to the construction of mechanical substitutes intended to supply the loss or defect of the palatine organs only, the surgical treatment will be entirely overlooked, and we therefore at once consider that peculiar defect, requiring the assistance of artificial palates or obturators. Passing over the cause originating this great want, we find the defect exhibited in the entire absence of the hard and soft palate, the velum and its appendages, most commonly under these circumstances attended with a considerable distortion in the relationship and relative condition of the dental circle, the alveolar processes, the spongy bones, and septum of the nose, depending greatly, however, upon the nature of the cause of this deformity, as also, if arising from disease, the age at which the destruction took place, from the greater or less flexibility of the bones on the ratio of the age of the subject. This condition of things presents many difficulties in the skillful adjustment of an obturator, as such appliances to become most useful, must almost always be made permanent fixtures by means of clasps, attached to the teeth, or by other means hereafter to be described; for this purpose, those teeth are selected which represent the greatest degree of firmness and power of resistance, unfortunately they are found too frequently presenting such an angle with the surface of the plate as to produce the difficulty named. The simplest form of palatine defect under consideration is, when there is a mere fissure of greater or less extent passing through the hard palate into the nose, but not extending posteriorly as far back as the soft appendages comprising the uvula tensor and levator palati, constrictor isthmi faucium, palato pharyngeus and azygos uvulæ, and not as far anteriorly as the alveolar border. We are particular in mentioning these muscles, as in some peculiar congenital deformities, they are merely separated along the line of the raphe, acting as great cords, contracting in deglutition with much power, and preventing greatly the restoration of these parts, either by surgical means or by the application of a plate with various appendages. The second in order is, when the fissure extends from the soft palate anteriorly, through the alveolar ridge, producing labio-leporinum, and requiring the addition of the missing incisor teeth to the obturator. The third for description is, when the defect involves these two in the manner as mentioned, with the entire separation of the soft palate, and fully exposing the nasal cavity, the posterior fauces and the orifices of the eustachian tubes, bringing the tonsil glands directly in sight. The fourth and last case to be described, to the foregoing combination is added the absence of all the teeth, the upper lip and the nose. It has always been a questionable point, whether the insertion of an obturator can be of any good in the restoration of the natural tone of voice, as well as to syllabic articulation, when the velum and connecting soft parts are destroyed, from the fact that these appendages to the palate perform such important offices in the production of all intelligent sound, and possess such immense variety of movement, as to prevent even a tolerable semblance in the construction of the most skillful substitute. In all the movements occurring in respiration, deglutition, and in almost all articulate sound, these perform a most prominent part, rendering it altogether probable that perfect restoration can never be obtained under any operation, whether surgical or mechanical; but• that most valuable assistance is often rendered, even in the worst species of deformity, is now equally beyond all doubt.

Considering the consequences attendant upon these malformations, is noted first the defect of speech, which unquestionably strikes a fearful blow against social happiness, and is esteemed the worst resulting evil. The labored awkwardness in taking food or drink. The disagreeable pitch of voice, always made in speaking and conversing.

It is found that the air passing from the lungs through the trachea reaches that part of the larynx, containing the true and false chorda vocales, it here receives from the expansion or contraction of these muscles with the proper positions of the whole vocal box, as it is termed, the quality of tone, which when properly received by the uvula and soft palate, is repeated upon the hard, and passed out of the mouth, the peculiar effect of tone is given by the conduct of the soft palate, not so much in the vocalization of sound, but particularly in the power of speech, in elevating or depressing the voice, so that it is found in those in whom the fissure passes from the anterior alveolus back through the hard and soft palate, that their little power of expression, (if they ever possess any,) is wholly gone under any attempt at modulation, or even extreme elevation of pitch whilst speaking, but must come to be ordinarily intelligible, in the unvarying monotony of force, volume and endurance, for the larynx has to perform the whole duty that in a natural state is so greatly assisted by the palates first, and the teeth and lips finally. In the absence of both the palates, there are certain tones and pitch and innumerable words which it is utterly impossible to be produced, which, in the absence of but one, remains only in a modified or disguised condition, for, to generalise, we find that all words which require the grave tones of the voice, are produced by the sound coming from the larynx and vibrating from off the soft palate, whilst those of the sharp pointed sound are vibrated upon the hard, easily procured in the first case by sounding a, o or i, and in the second, e, m, n, &c. It will be readily seen, under this exposition, the hopelessness of attempting to remedy this great defect entirely, except in the mere absence of a perfect hard palate, which can be done in all cases with ease and certainty, but when the destruction of the whole, or a part of the soft and hard palate occurs, its relief must be regarded as resting in part only in the domain of the most enlarged skill, at least so it must rest at present. Deglutition, fortunately, is more easily accomplished under these circumstances, and nature struggling in her might overcomes, to a great extent, these obstacles to complete mastication, for such patients will generally be found to eat almost anything and everything pertaining to the ordinary food, with ease, or at least without complaint, but of course it is an acquirement after all, for the food to be well triturated must need the natural assistance of the palate, and an equal necessity must there be for a channel of suitable dimensions through which to carry the food to the æsophagus, but they do manage to eat and drink under many distressing inconveniences.

We are led to regard Ambrose Paré, as the first who constructed an appliance to remedy this evil, which he did in the year 1585; rude though it was, it has doubtless been the foundation of all after construction, even up to the present day, which consisted of a plate fitting over the orifice, and there suspended by means of an attached sponge entering the nasal cavity. Fauchard followed with the improvement of wings turning upon pivots, in place of the sponge. Bourdet next suggested the plate to be sustained by silk ligatures, made fast to the teeth, which was again finally improved upon by M. Delabarre in the addition of metallic clasps, in place of the ligatures, and of a drum, forming a base or floor to the nose, and thus preventing disagreeable accumulations, as well as the regurgitations of fluids from the mouth into the nose. This instrument is made with facility by taking the impression of the parts, including the whole alveolar ridge, in soft wax, which will be found preferable to the use of any other material, having care in withdrawing it, that the part which has filled the fissure be not displaced by undue pressure in either direction; it is desirable that this portion of the impression should come in gentle contact with the remaining portions of the nasal cavity, so that the drum should, in fact form a complete floor of the nose, and slightly touch all surfaces, to the exclusion of even the air in breathing, for it will be found that all the parts entering into the function of speech, have become trained in forming sound, the omission of which has been almost altogether through the palatine fissure and nose, to correct which, is the grand object in the efforts to restore to these parts their legitimate effects of course, then, the continuance of the means by which this deformity has been permitted, can only eventuate the same result, and to prevent which an obturator is desired. Having obtained the impression of the mouth, and the plaster model, you proceed to form a continuation of what would have been the natural roof or palate of the mouth, by filling up the depression in the plaster model even with the edge of the fissure, the counter-models being taken, your plate is now swaged up, and clasps adjusted in the mouth, you must now proceed to make the drum to fill the aperture, to do this, the filling, which was placed in the plaster model, is now removed, carefully marking the point of its connection with the plate which has been made, so as to determine the length of the drum required, and its exact adjustment to the plate, you now again take metal dies, and strike up this drum to the size as directed, and finish its adjustment to the plate, it is now to be tried in the mouth, and will be found to fit accurately, if care has been had with each step; great nicety is now required to solder this drum upon the plate in its exact position, to do which, it must be fixed in its position in the mouth, by the following method, attach it to the plate by wax placed upon the inner side, as near as can be judged correct by your plaster model, this wax should be as warm as the parts will bear, so that any readjustment can be made with facility, when placing it in the mouth; for the greatest care must be had that the drum is not placed in any way out of its true position, for irritation would most certainly follow such a result. Having satisfied yourself that its position is as it should be, withdraw it and mark with a sharp instrument the exact position of the drum upon the plate, so that it can be removed, the wax cleansed off, the surface scraped, and the two soldered together precisely as it was placed. It is preferable that the drum should be held to its position for soldering by means of wire wrappings, in place of holding one-half at a time with plaster and sand, thus the under surface of the plate needs only the protection of the plaster and sand, or better still, solder it without protecting it with plaster, provided the wire will not draw the plate from its required shape, and the drum can be so soldered on at one heating. If teeth are to be appended to the piece, they should now be adjusted in the mouth, backed and soldered in the usual manner, care being had that the heat should be thrown off from over the drum by means of plaster and sand, or perhaps asbestos used instead of the sand, to prevent it from becoming unsoldered. The next species of obturators claiming attention, is that, in which the whole of the hard and soft palates, uvula and its appendages are to be supplied.

The first great point, is to obtain a correct impression of the mouth, which under these circumstances presents an incredible large cavern, and leads you at once to infer that the substance, of sufficient size to fill it, cannot be withdrawn through the lips, but fortunately this can generally be done, when sufficient patience and skill is exercised in the attempt. It is necessary, however, that the impression cup should have been made expressly for each case; so that as little amount of wax is used as possible, and that it is so placed as to present the least difficulty, to adapt or press it to each and every part, to withdraw it; or it may have to be turned in many directions, so as to remove it from the mouth without displacing the wax, or disfiguring its surface by contact with the teeth. To do this, avoid all haste, taking plenty of time and having the most watchful care.

Cut 1.

Having given, in the foregoing, a general description, we will now illustrate a few of the cases; the first cut, 1, represents the most simple kind of obturator, where there is a mere fissure in the palate of small dimensions, but which produces great defects in all processes in which this part of the mouth performs a part, but principally in speech and in deglutition. It requires, as is represented, a simple plate accurately adjusted over and around the fissure, and attached on both sides to two healthy teeth, those that present the greatest degree of firmness must be selected, and the clasps made as the teeth will allow, and most accurately adjusted, for if there is the least rocking or unequal pressure, fatal results must follow, the piece finally becoming useless, and destroying the teeth by which it is supported. If the plate has been well fitted, no unpleasant accumulation can take place, that which which will occasionally gather immediately about the fissure, being ejected through the nose. In all cases, great care must be had to prevent causes for irritation about the clasps or edges of the plate by undue pressure, from the fact previously mentioned of the proneness, in all cases suffering from this condition, to take on irritation and succeeding inflammation.

Cut 2.

Cut 2, represents the same plate with a drum affixed and is used where the fissure is very large, and when the velum remains, but only as a muscular band stretching across to either side, permitting fluids and particles of food to pass over it, and were it not for the drum, into the nasal cavity. The drum fills up the fissure, it upper surface forming the floor of the nose and coming in easy contact with the septum nasi, the posterior edge fitting lightly against the velum, which rests upon that portion of the plate which projects towards the fauces. The circumference of the drum should fit easily against the surrounding surfaces of the fissure. Care must be taken that the posterior edge of the plate be guarded against irritating the soft muscle working at this point, by soldering a rim of wire to it, thus presenting a smooth, round edge, and as each case becomes more complicated, greater precaution must be taken to prevent the liabilities before mentioned, from improper adjustment of each part and of the whole plate.

Cut 3.

Cut 4.

Cuts 3 and 4 are obturators inserted in a case, the deformity having occurred from disease, all the parts within the mouth being in a constant state of irritation. The syphilitic disease had destroyed the entire hard and soft palates, leaving nothing but the alveoli containing the teeth, and the muscles on either side of the fauces. It was first thought expedient to try the obturator invented by Dr. S. P. Hullihen, in which a simple plate has attached to it a valve, supported in its position towards the nares by means of a spiral spring and slide, working within loops for this purpose, fastened upon the plate, and allowing the valve by this means to be adjusted in its position with the tongue, and which may be found well described in vol. 2, of this Journal, N. S. It was found, however, inapplicable as the muscles would not allow a valve of the size necessary to fill the aperture to remain there without great irritation, for in the act of swallowing they would press upon it with such force as to give pain, producing violent retching and coughing. The plan then adopted was that represented in the above cuts, in which the two valves were movable by the agency of the spiral springs, as seen in cut 3, portraying the lower surface, and 4, the upper. The main plate has a second plate attached, running upwards and backwards at an angle of about 40°, the springs are soldered at one end on the upper surface, and extending back through this second plate, having little arms extending through it to sustain the valves projecting on either side, and with small narrow apertures for the necessary motion in their movements. By this means, the space was well filled to the exclusion of passing air, food and fluids, the muscles in contracting, found no opposing obstacle, the valves closing and unclosing with the existence of their slight pressure.

Cut 5.

The cuts, although poor, will give a tolerable representation; suffice it to say this method has fully answered the desired end.

Cut 5, is an obturator complicated with both teeth and drum as the case may demand, requiring no particular description, the previous matter having fully comprehended the case as represented, as is also the case in the following 6, 7, 8, 9. [See next page.]

These latter, as will be perceived, carry a drum, whose upper surface represents the irregularities of the fissure, being requisite in cases where no teeth remain, and its support is had from its perfect adaptation to all the parts. But, as before remarked, no rules or guides can be given which will be found applicable to every case, each case will differ in many particulars, requiring more or less ingenuity of the operator. But

Cut 6.

Cut 7.

Cut 8.

Cut 9.

enough has been said to admit of general appropriation of many points, and enable the skillful to accommodate the majority of patients demanding palatine obturators.

### ARTICLE VI.

*Lusus Naturæ—A Curious Case of Anomalous, and Non-development of Teeth.* By C. T. Cushman, D. D. S., Columbus, Ga.

## Figures and Tables

**Cut 1. f1:**
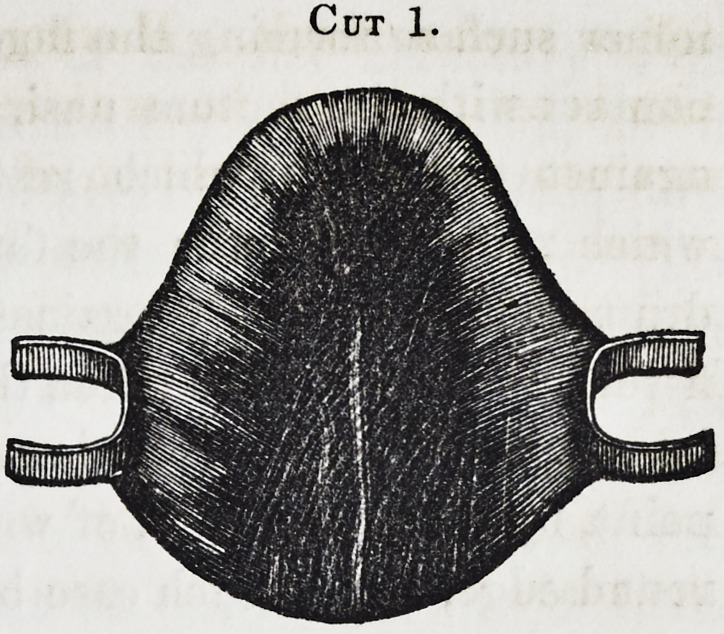


**Cut 2. f2:**
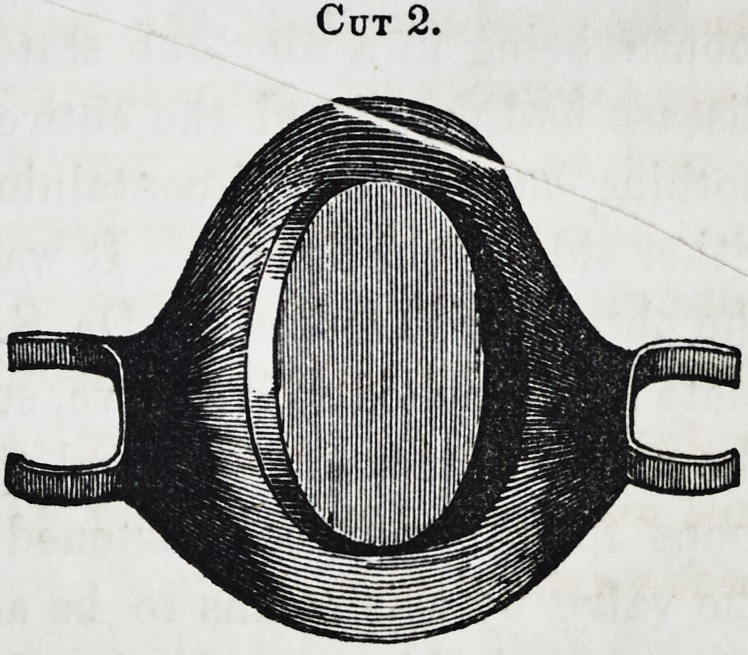


**Cut 3. f3:**
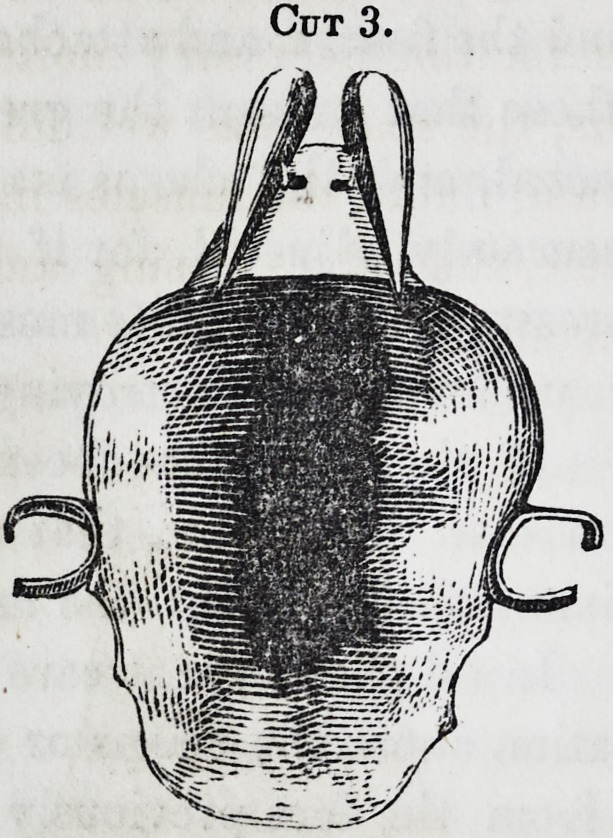


**Cut 4. f4:**
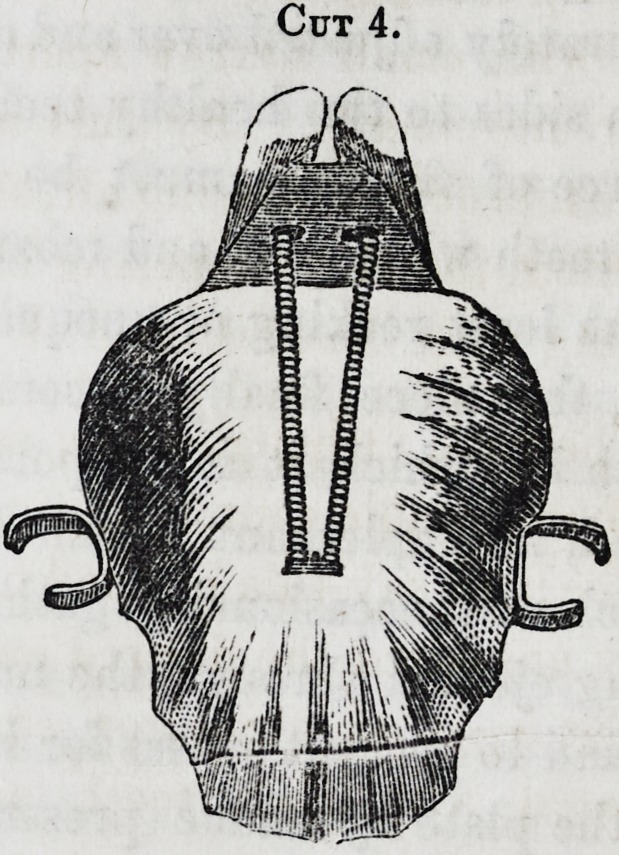


**Cut 5. f5:**
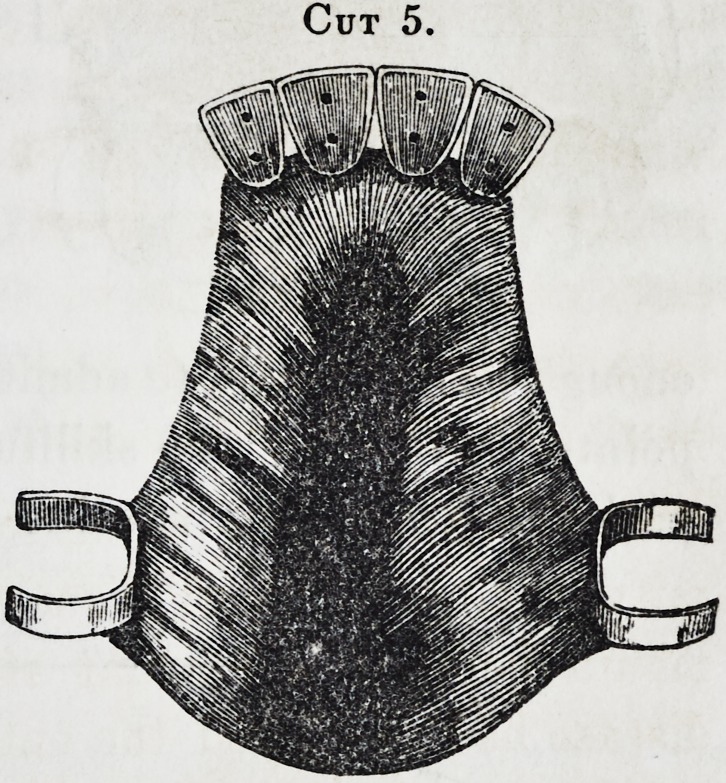


**Cut 6. f6:**
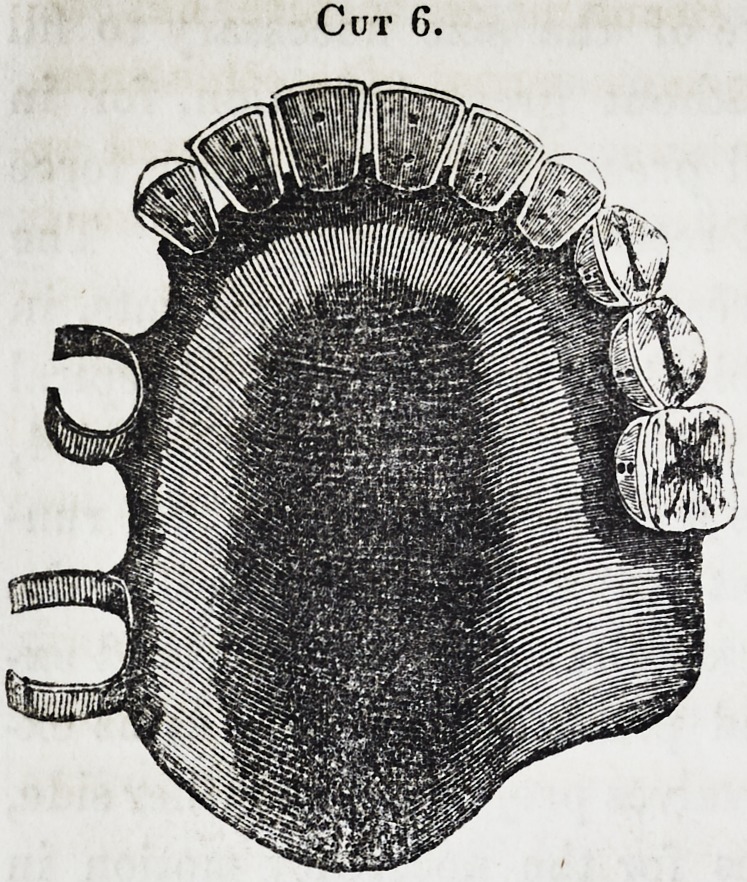


**Cut 7. f7:**
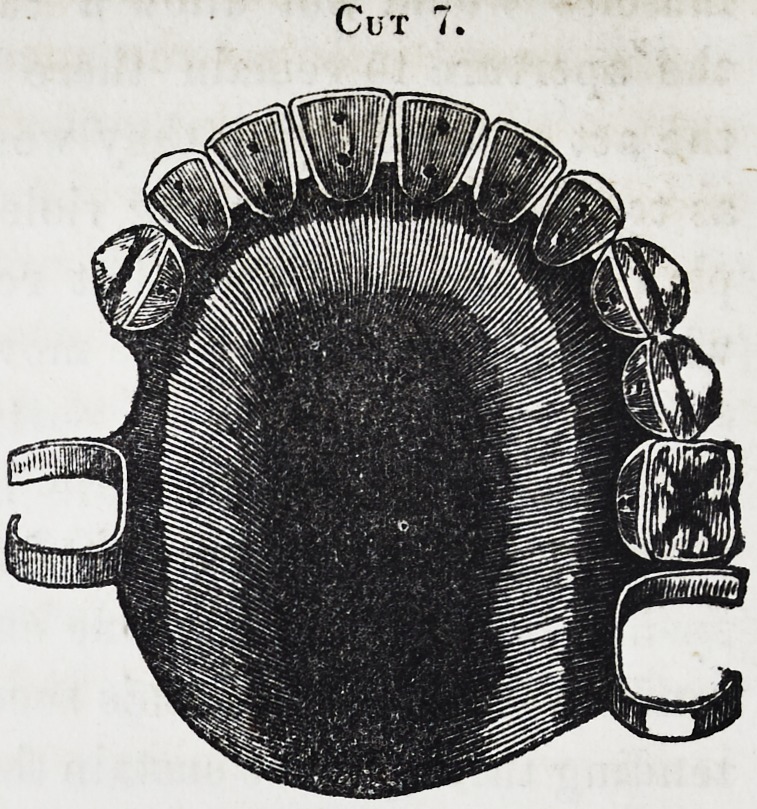


**Cut 8. f8:**
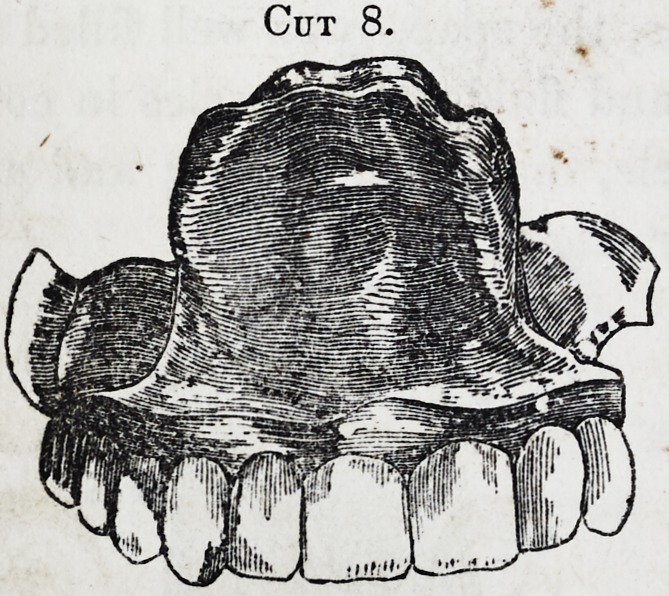


**Cut 9. f9:**